# Supplementary figures and images for: Disentangling direct and indirect effects of local temperature on abundance of mountain birds and implications for understanding global change impacts
Source: PeerJ. 2021 Dec 3;9:e12560. doi: 10.7717/peerj.12560 (PMC8647716; doi:10.7717/peerj.12560)

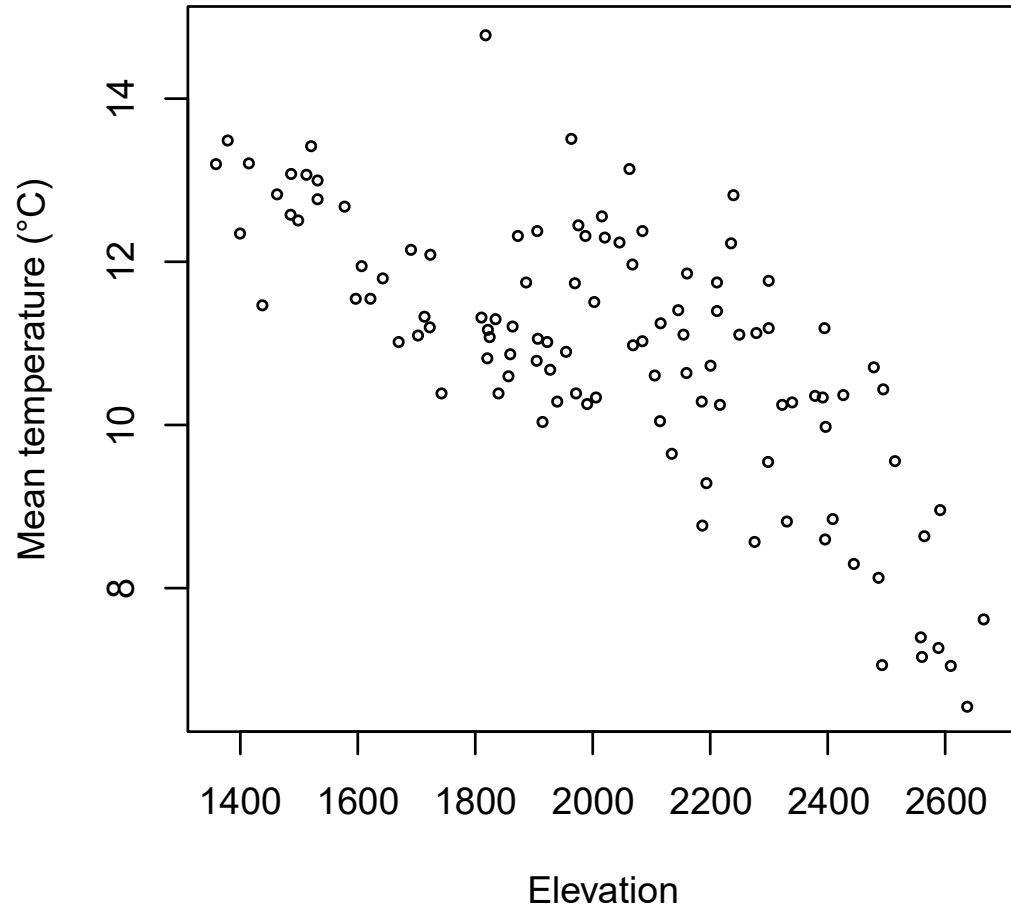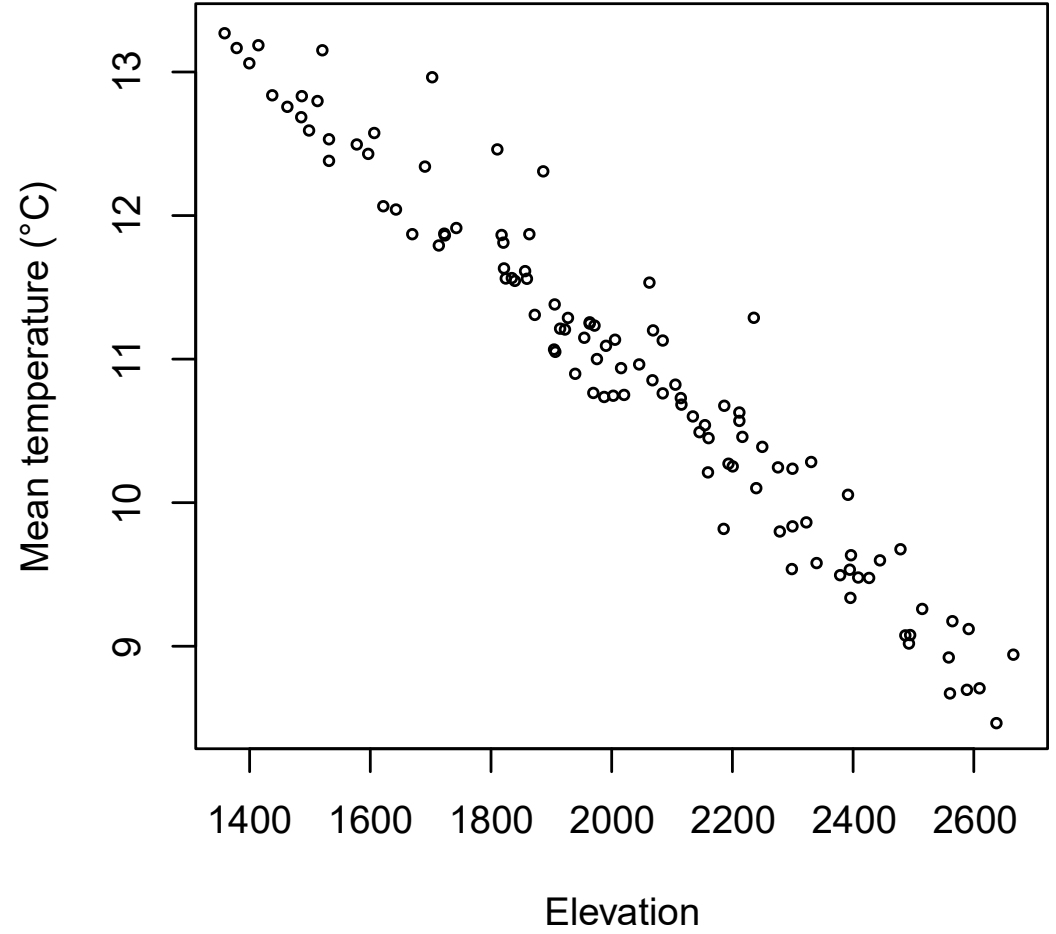

Supplement: Supplemental Information 2 — Elevation and aspect jointly explain 56% of variation in mean temperatures according to multiple linear regression (mean temperature ~ elevation + aspect), and both show a significant effect (elevation: β = −1.3, p < 0.0000; aspect: β = −0.3, p = 0.007). [file peerj-09-12560-s002.pdf]
