# Supplementary material for: Disentangling direct and indirect effects of local temperature on abundance of mountain birds and implications for understanding global change impacts
Source: PeerJ. 2021 Dec 3;9:e12560. doi: 10.7717/peerj.12560 (PMC8647716; doi:10.7717/peerj.12560)
Supplement: Supplemental Information 4 — Spatial autocorrelation in model residuals for each species, according to Moran’s I test. [file peerj-09-12560-s004.docx]

Table 1S: Spatial autocorrelation in model residuals for each species, according to Moran’s I test.

| Species | Moran’s I | p |
| --- | --- | --- |
| *Anthus spinoletta* | 0.148 | 0.00086 |
| *Certhia familiaris* | 0.025 | 0.46856 |
| *Erithacus rubecula* | 0.119 | 0.00672 |
| *Fringilla coelebs* | -0.005 | 0.92602 |
| *Lophophanes cristatus* | 0.043 | 0.25851 |
| *Oenanthe oenanthe* | 0.187 | 0.00003 |
| *Periparus ater* | 0.182 | 0.00007 |
| *Phylloscupus collybita* | 0.414 | < 0.0001 |
| *Poecile montanus* | 0.065 | 0.11781 |
| *Prunella modularis* | 0.049 | 0.22513 |
| *Pyrrhula pyrrhula* | 0.183 | 0.00006 |
| *Regulus regulus* | 0.222 | < 0.0001 |
| *Sylvia atricapilla* | 0.233 | < 0.0001 |
| *Turdus torquatus* | -0.009 | 0.99966 |
| *Turdus viscivorus* | -0.037 | 0.53382 |
